# Evaluation of Biocontrol Activities of *Streptomyces* spp. against Rice Blast Disease Fungi

**DOI:** 10.3390/pathogens9020126

**Published:** 2020-02-15

**Authors:** Mathurot Chaiharn, Teerayut Theantana, Wasu Pathom-aree

**Affiliations:** 1 Division of Biotechnology, Faculty of Science, Maejo University, Chiang Mai 50290, Thailand; mathurot@mju.ac.th; 2 Department of Biology and Biotechnology, Faculty of Science and Technology, Nakhon Sawan Rajabhat University, Nakhon Sawan 60000, Thailand; tee_yai18@hotmail.com; 3 Department of Biology, Faculty of Science, Chiang Mai University, Chiang Mai 50200, Thailand; 4 Center of Excellence in Microbial Diversity and Sustainable Utilization, Faculty of Science, Chiang Mai University, Chiang Mai 50200, Thailand

**Keywords:** biological control, *Oryza sativa*, rice blast disease, *Pyricularia* sp., Streptomyces sp., plant growth promotion, greenhouse condition

## Abstract

Rhizosphere bacteria can positively influence plant growth by direct and indirect mechanisms. A total of 112 bacterial strains were isolated from the rhizosphere of rice and tested for plant beneficial activities such as siderophore production, cell-wall-degrading enzyme production, hydrogen cyanide (HCN) production and antifungal activity against rice blast disease fungus. The actinomycetes count was 3.8 × 10^6^ CFU/g soil. *Streptomyces* strains PC 12, D 4.1, D 4.3 and W1 showed strong growth inhibition of blast disease fungus, *Pyricularia* sp. (87.3%, 82.2%, 80.0% and 80.5%) in vitro. Greenhouse experiments revealed that rice plants treated with *Streptomyces* strain PC 12 recorded maximum plant height, root length and root dry weight compared to the control. Taxonomic characterization of this strain on the basis of 16S rRNA gene sequence led to its identification as *Streptomyces palmae* PC 12. *Streptomyces palmae* PC 12 may be used as biofertilizer to enhance the growth and productivity of commercially important rice cultivar RD6 and the biocontrol of blast disease fungus.

## 1. Introduction 

Rice (*Oryza sativa*) is an important food crop and it is the staple diet of people around the world, especially in Asia. However, rice is susceptible to diseases by phytopathogenic fungi that cause crop yield losses. Some of them also produce toxic compounds which are harmful upon consumption [1,2]. The rice blast fungus *Pyricularia* sp. is one of the most devastating airborne pathogens [3]. The fungal spores attach to the host surface by mucilage secreted from the spore tip. The germinated spores produce an extracellular matrix (ECM), and firmly attach to the plant surface [4]. The attached spores develop a germination tube, an aspersorium and a penetration peg, and complete infection by signal exchange with the host plant via ECM [4]. The ECM of *Pyricularia* sp. helps the fungal spore adhesion to the cellulose membrane and suppressed disease occurrence in the plant cell [5,6]. The fungus can infect rice plants at any growth stage and infects the aerial parts of rice including leaves, nodes, stems and panicles [2,3,7]. The mycelium may survive within the tissues of embryo, endosperm and glumes. Rice blast symptoms include leaf blast, node blast, collar rot, neck rot and panicle blast, which manifests as grayish/brownish spots or lesions as well as the withering of leaves [2,3,8]. The fungus infects the roots of the rice plant and spreads to the aerial tissues, causing rice blast diseases [9]. Yield loss due to rice blast ranges from approximately 10–30% in various rice-producing countries and can increase by up to 50% during disease outbreak [2,8,10]. Many fungicides have been used against blast disease, i.e., azoxystrabin, benomyl, carbendazim, carpropamid, dithiocarbamate, edifenphose, fenoxanil, tiadinil, tricyclazole, pyroquilon, probenazole, iprobenfos, isoprothiolane, metominostrobin and propiconazole [3,11,12]. However, the synthetic fungicides cause environmental pollution, residual problems, the development of pesticide resistance, soil quality reduction and damage to natural ecosystems [11,12]. The control of blast disease using fungicide adversely affects both the environment and soil microbiota [13]. Additionally, human exposure to pesticides may be harmful to health, causing skin irritation, headache, eye irritation and shortness of breath due to the exposure to various pesticides during mixing and spraying [14], and biological processes such as the reproductive system and hormone levels [15]. 

Current strategies used to control rice blast disease include the use of disease-resistant rice varieties and synthetic fungicides. However, *Pyricularia* sp. develops new strains rapidly, resulting in a breakdown in rice resistance [12,13]. Nowadays, natural products which are safe for the environment and have low toxicity to living organisms are gaining interest as important sources for the development of fungicides. *Streptomyces* are natural soil-dwelling bacteria that have been largely used as biological control agents [16]. *Streptomyces* species have excellent capacities to produce a variety of bioactive compounds, such as antibacterial, antifungal, antiviral, anticancer and antioxidant properties [16]. Some antibiotics produced by *Streptomyces* have been used as fungicides for the control of rice blast, i.e., Blasticidin-S and Kasugamycin [12,16,17]. For example, Kasugamycin was safely used to protect rice plants against blast disease with low mammalian toxicity and a lack of phytotoxicity toward rice plants and most crops [17]. Oligomycin A, Rapamycin and Pyrroles (Pyrroles [1,2-a] pyrazine-1,4-dione, hexahydro) were found to control the development of rice blast [18]. *Streptomyces* spp. can be an alternative to synthetic chemical fungicides and used as a biocontrol agent. This biological approach to plant disease management offers a better alternative to control blast disease due to its safety for human use and the environment.

The use of *Streptomyces* as biocontrol agents of rice blast disease is still limited, especially in Thailand. Boukaew and Prasertsan reported the isolation of *S. philanthi* RM1-138 from rhizosphere soil of chili pepper in southern Thailand [19]. This *Streptomyces* showed strong in vitro antifungal activity (88.73% inhibition) on the mycelial growth of *Magnaporthe oryzae* (*P. oryzae* PTRRC-18). Other studies also indicated the potential of *Streptomyces* species to control the disease [12,16,20,21,22,23]. The present study aimed to isolate antagonistic *Streptomyces* from rice rhizosphere soil and investigate their plant growth promotion and antifungal activity against rice blast fungus *Pyricularia* sp. in vitro and under greenhouse conditions.

## 2. Results 

### 2.1. Isolation of Rhizospheric Actinomycetes

**Figure 1:**
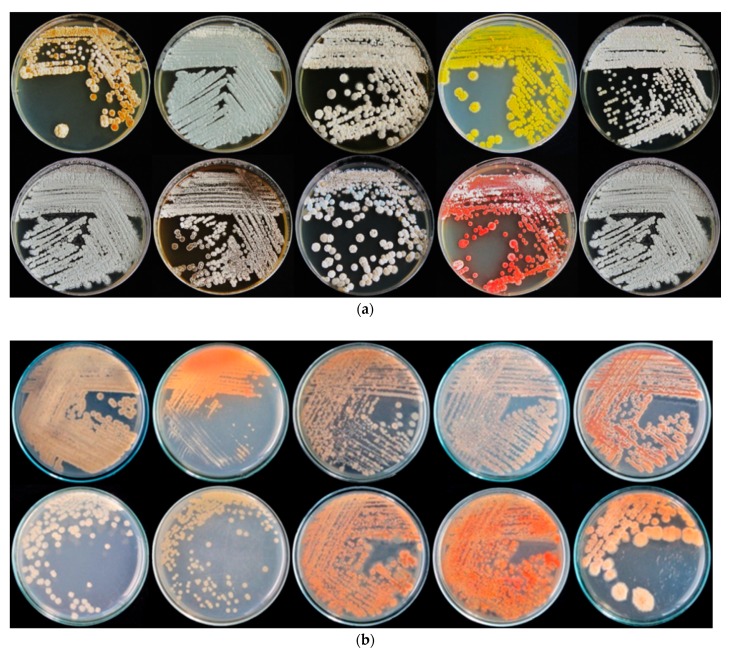
Characteristic of the aerial (**a**) and substrate mycelium (**b**) of some rhizospheric actinomycetes isolated on ISP-2 medium after being incubated at 30 °C for 14 days.

A total of 112 actinomycetes were isolated from the rhizospheric soil of rice in Maerim district, Chiang Mai province, Thailand (18° 56′ 17.2104” N and 98° 53′ 1.7520” E). Isolated actinomycetes showed well-developed substrate mycelium, were filamentous branched, and most aerial mycelium appeared floccose, granular or powdery. Colonies with characteristic features, such as a powdery appearance and color ranging from white or gray to pinkish and yellowish, were selected. The isolates obtained formed colored tough, leathery and filamentous colonies that were hard to pick from the culture media, as a characteristic of genus *Streptomyces,* and produced colored pigments. Preliminary assignment of *Streptomyces* according to color of aerial mycelium and presence of soluble pigments is shown in Table 1. Ten main classes of color were observed with grey, brown, white-brown and white as the main colors (Table 1 and Figure 1). Cell wall composition analysis of actinomycetes using thin-layer chromatography (TLC) revealed a type I cell wall with *LL*-DAP isomers.

### 2.2. Isolation of Blast Disease Fungi from Rice Leaves and Pathogenicity Test 

**Figure 2:**
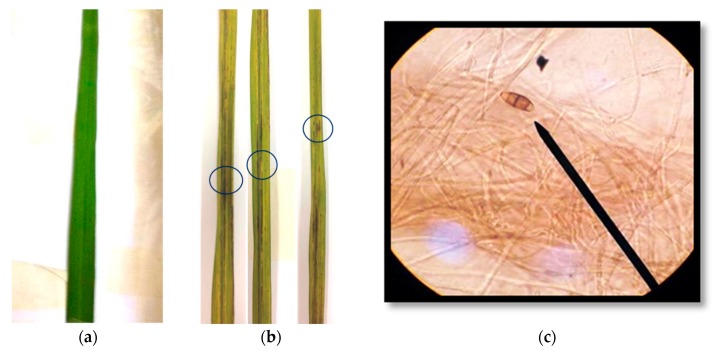
Typical blast symptom on rice (*Oryza sativa* L.) cultivar RD6 leaves 14 days after inoculation (**a**) Healthy leaf, (**b**) infected sick leaves and (**c**) *Pyricularia* sp. WPP09 spores at magnification 400 X by Compound light microscope (Olympus CX 23).

Fifteen isolates of *Pyricularia* sp. were isolated from the diseased leaves of rice (*Oryza sativa* L.) cultivar RD 6 and cultured on a potato dextrose agar (PDA) medium at 28 °C for 7 days for 10 days. Morphological characterization on the PDA plate showed that all fungal isolates developed a white, light gray or dark gray mycelium that formed concentric rings on the growth medium. Microscopic observation showed pear-shaped conidiospore with a tail-like structure at the posterior end. The conidia consisted of 2–3 septations with a hyaline brownish color (Figure 2c). Pure culture of isolated *Pyricularia* strains were inoculated onto rice (*O*. *sativa* L.) cultivar RD 6. *Pyricularia* strain WPP09 reduced the plant height and weight of RD 6 and induced blast disease symptom compared to control plants. Fourteen days after inoculation, 80% diseased plants were recorded (Figure 2b) in *Pyricularia* inoculated plants, whereas disease was not developed in un-inoculated plants (Figure 2a).

### 2.3. Plant Growth Properties of the Actinomycete Isolates

In the present study, some isolated strains were positive for plant growth promotion and biocontrol activities. Only strains with a significant antifungal activity in vitro against *Pyricularia* sp. were shown in Table 2. *Streptomyces* strain PC 12, *Streptomyces* strain D 4.1, *Streptomyces* strain D 4.3 and *Streptomyces* strain W1 showed maximum growth inhibition of *Pyricularia* sp. (87.3%, 80.0%, 82.2% and 80.5%) in a dual-culture plate. Based on percent inhibition of the pathogen, four actinomycetes strains, namely W1, PC 12, D 4.1 and D 4.3, were selected for greenhouse experiment (Table 2). All strains were positive for HCN production, had strong siderophores production and produced cell-wall-degrading enzymes, thus indicating their potential for antifungal activities (Table 2). Siderophore production was screened by observing the change in color from blue to orange in CAS agar plates. Twenty-seven isolated strains were siderophore positive with varying intensities of orange zones. Strain D 4.1 (52.3 mm), strain D 4.3 (51.4 mm), strain PC-117 (50.7 mm) and strain PC-94 (50.2 mm) exhibited the maximum siderophore producer on the CAS agar plate (Table 2). All isolates were positive for protease activity with a zone diameter ranging from 24.2 mm to 73.7 mm. Nine isolates were positive for cellulase, with strain D 4.1 as the best producer (70.8 mm), followed by strain D 4.3 (69.5 mm) and strain PC-55 (64.2 mm). Chitinase activity was positive for seven strains. Maximum chitinase activity was observed in strain D 4.1 (57.4 mm), followed by strain D 4.3 (48.5 mm) (Table 2). 

### 2.4. Greenhouse Experiment

#### 2.4.1. Pathogenicity Test of Selected Streptomyces 

We evaluated the pathogenicity of four selected *Streptomyces* strains, W 1, PC 12, D 4.1 and D 4.3, on rice RD 6. No sign of abnormalities, such as lesion formulation or wilting, were observed in seedlings at 14 days. 

#### 2.4.2. Evaluation of Streptomyces against Pyricularia sp. under Greenhouse Condition

Four Streptomyces (strain W 1, strain PC 12, strain D 4.1 and strain D 4.3) were selected and screened under greenhouse conditions. The initial disease symptoms appeared five days post-inoculation (dpi). Typical symptoms of blast on the rice leaves were diamond-shaped lesion with a grey or white center and brown border (Figure 2). Symptoms of blast increased daily from 4 dpi (day post inoculation) and reached their peak on 60 dpi in the inoculated rice plant. The progression of disease in terms of disease severity varied among the treatments (Table 3). Disease severity in pathogen-inoculated control plants was 87.5% at 60 dpi, while in pathogen + *Streptomyces*, it ranged from 51.9% to 31.4%. PC 12-inoculated plants showed the lowest disease severity (31.4%). No disease development was observed in the untreated control. Compared to pathogen-inoculated plants, Pathogen + *Streptomyces* PC 12-inoculated plants had a significantly lower disease severity (35.8%) compared to the control (Table 3). In addition, treatments with *Streptomyces* PC 12 and *Streptomyces* D 4.3 showed slower blast disease progression than the other treatments (Table 3).

#### 2.4.3. Plant Growth Promotion effect 

Lesion length and number of dried leaves

Plants treated with *Streptomyces* strain PC 12 showed the lowest number of leaf lesions and dried leaves compared to the other tested isolates. All isolates significantly reduced the disease compared to the control. Strain PC 12 showed the lowest mean lesion length (0.55 cm) and number of dried leaves (3.10 leaves/plant) (Table 3). These low disease parameters indicate the healthy status of PC 12-inoculated plants. 

Plant height 

All tested strains enhanced the plant height compared to control (Table 3). At 60 days, the highest plant height (76.93 cm) was recorded in the treatment of *Streptomyces* strain PC 12. This was followed by the treatment of *Streptomyces* strain D 4.3 (52.69 cm). The control plant treated with pathogen showed the lowest height. In addition, the height of rice in PC 12-inoculated plants was almost double that of un-inoculated plants, which indicated that rice was in a healthy condition. 

Root length and root dry weight 

In general, the root length of plants from all treatments with selected Streptomyces was longer than the control (Table 3). The longest root length (41.60 cm) was recorded in rice inoculated with *Streptomyces* strain PC 12. Similarly, the dry weight of root was enhanced in all treatments with selected *Streptomyces*. Again, *Streptomyces* strain PC 12 yielded the highest root dry weight of 2.68 g. Plants treated with the fungal pathogen showed the lowest dry weight (1.88 g) (Table 3). These observations suggested the healthy status of PC 12-inoculated plants compared to the pathogen-treated control. 

Number of tiller

The average number of tiller was significantly enhanced by all tested *Streptomyces* PC 12 in pathogen + *Streptomyces*-inoculated plants. *Streptomyces* PC 12 -inoculated plants yielded the highest number of tiller (19.4) over pathogen-inoculated control and untreated control plants, suggesting that the rice was healthy.

### 2.5. Rhizospheric Colonization by Streptomyces 

At the beginning of the experiment, the actinomycetes isolates had an average cell number of 3.0 × 10^6^ CFU/mL. After the selected *Streptomyces* strains were applied to the plants for 2 months, the amount of *Streptomyces* strain PC 12 decreased to 4.5 × 10^4^ CFU/mL (Table 4). Other *Streptomyces* strains also showed a decreasing trend. The persistence of the strains in the rhizosphere may account in part for the varied level of disease suppression between different *Streptomyces* sp. treatments.

### 2.6. Identification of Streptomyces PC 12

**Figure 3:**
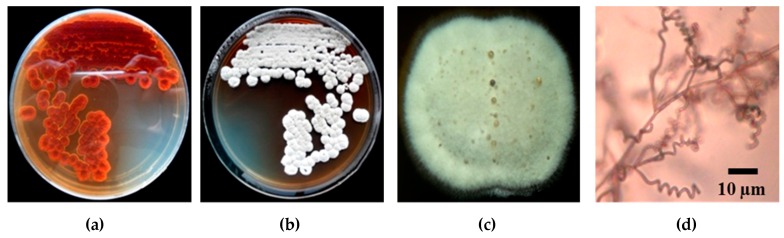
Macroscopic determination and Microscopic determination of *Streptomyces* strain PC 12 (**a**)*Streptomyces* strain PC 12 culture on ISP2 medium at 28 °C for 7 days showed substrate mycelium (**b,c**) *Streptomyces* strain PC 12 culture on ISP2 medium at 28 °C for 7 days showed white aerial mycelium (**d**) Photomicrograph of spiral spore chains of *Streptomyces* strain PC 12 under Compound light microscope.

**Figure 4:**
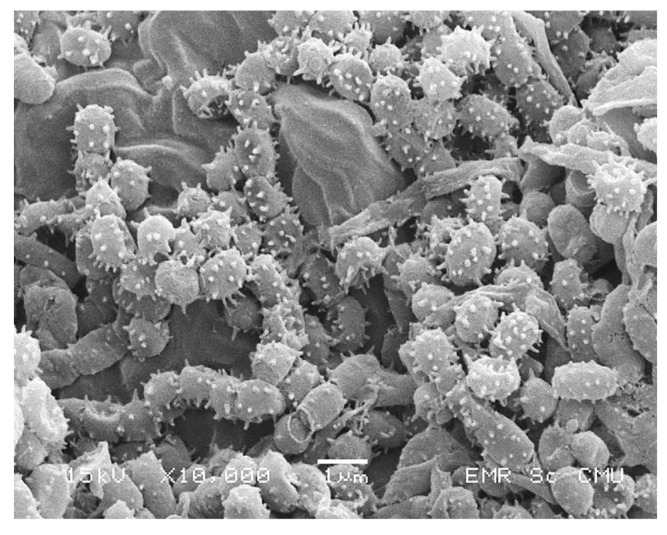
Scanning electron micrograph of the spore chain and the spiny spores of *Streptomyces* strain PC-12 culture on the ISP 2 agar for 14 days.

Strain PC 12 was observed to grow on a variety of ISP agar, including yeast-extract-malt-extract agar (ISP-2), oatmeal agar (ISP-3), inorganic salts/starch agar (ISP-4) and peptone/yeast agar (ISP-6). Aerial mycelium color was white and substrate mycelium was grey on ISP-2 media (Figure 3a,b). Melanin pigments were not observed on any of the media tested. Spiral spore chains were observed under light microscope (Figure 3d). Spore chain and spore surface morphology of strain PC 12 was determined by scanning electron microscope (SEM). Spores were spherical with a spiny spore surface (Figure 4). TLC analysis of whole-cell hydrolysates of strain PC 12 showed *LL*-diaminopimelic acid (*LL*-DAP) (data not shown). 

**Figure 5:**
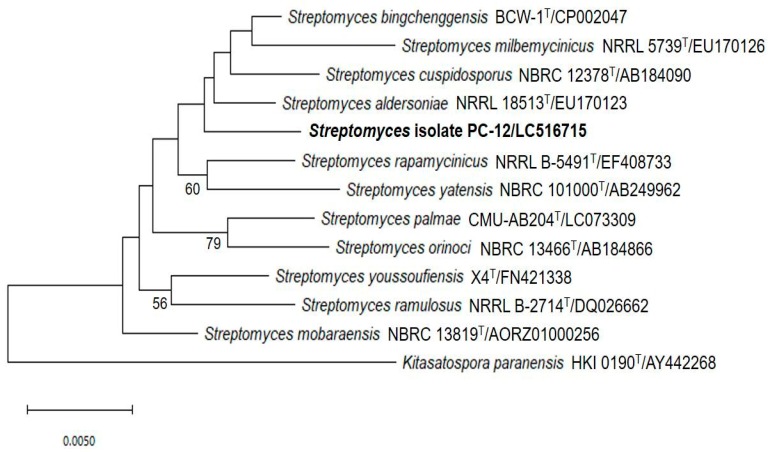
Neighbor-joining tree showing the position of strain PC 12 compared to its related *Streptomyces* species. Numbers at the nodes indicate the percentage of bootstrap values based on 1000 replicates. Only values greater than 50% are shown.

Based on chemical, cultural and morphological characteristics, strain PC 12 belongs to the genus *Streptomyces* according to Nonomura’s key [24]. The 16S rRNA gene of strain PC 12 was sequenced and compared with related *Streptomyces* species deposited in the GenBank database which indicated that, phylogenetically, strain PC 12 belonged to genus *Streptomyces*. BLAST analysis showed that strain PC 12 was closely related to *Streptomyces palmae* CMU-AB204^T^, with 98.82% similarity. Phylogenic analysis based on 16S rRNA gene sequences using the neighbor-joining methods was shown in Figure 5. Strain PC 12 formed an independent clade separated from *S. palmae* ^T^ CMU-AB204^T^.

## 3. Discussion

The present study was designed to isolate strains of actinomycetes from rice rhizosphere in an attempt to investigate their potential to control rice blast fungus *Pyricularia* sp in vitro and under greenhouse conditions. In this study, 112 isolates were recovered. Morphologically, all isolates were assigned to the genus *Streptomyces* [24]. Actinomycetes are commonly found in rhizosphere soil, especially members of the genus *Streptomyces* [16]. These microorganisms are well documented as potential candidates to inhibit the growth of several fungal plant pathogens including *Pyricularia* spp. [12,16]. Rice blast caused by *Pyricularia oryzae* is the most serious disease in all rice-growing regions worldwide [2,3]. In the present work, fifteen fungal strains were isolated from disease leaves of rice RD 6 and showed a typical morphology of *Pyricularia* species according to Mew and Gonzales (2002) [25] and Bussaban et al. [26]. The conidial shape can be used to differentiate *Pyricularia* from closely related genera and the spore morphology is consistently correlated with phylogenetic analysis [26]. 

Controlling rice blast disease has become a major concern, as it impacts rice productivity worldwide [2]. In this study, *Streptomyces* PC 12 was found to be capable of suppressing rice blast disease and promoting plant growth. When tested in vitro, strain PC 12 greatly inhibited *Pyricularia* WPP009 mycelial growth (87.3%), suggesting its capability of inhibiting the rice blast pathogen (Table 2). Our results are supported by with other works which reported the potential of *Streptomyces* strains as biocontrol agents against *Pyricularia* spp. under laboratory conditions*. S. vinaceusdrappus* was isolated from sediment of Loktak lake in India [20]. It showed 53.5% growth inhibition against *P. oryzae* MTCC1477. Simialrly, *S. philanthi* RM-1-138 isolated from chili pepper rhizosphere soil of southern Thailand could inhibit growth of *P. oryzae* PTRRC-18 in vitro [19].

Some studies also reported the evaluation of *Streptomyces* as a biocontrol agent for *Pyricularia* spp. under greenhouse or field experiments. For example, *Streptomyces* UPMR54 was reported to reduce rice blast disease by 67.9% and promoted rice growth and yield [21]. Antifungal compound, SPM5C-1 from *Streptomyces* strain PM5 completely inhibited mycelial growth of *P. oryzae* in vitro at concentrations of 25 μg/ml [22]. In addition, blast disease was reduced by 76.1% in greenhouse experiment at 500 μg/ml of SPM5C-1. Recently, foliar treatment of *Streptomyces hygroscopicus* OsiSh-2 culture filtrate showed 23.5% and 28.3% disease reduction of *P. oryzae* in rice seedlings under greenhouse and field trials [23]. In the present study, it was evident that *Streptomyces* strain PC 12 efficiently reduced blast severity from 87.5% to 31.4% in pathogen-infected plants under greenhouse conditions (Table 3). 

The ability of potential biocontrol agents to promote plant growth is considered as an added advantage. *Streptomyces* PC 12 inoculation was found to enhance rice growth, as indicated by plant height, root length, root dry weight and number of tiller (Table 3). It is known that *Streptomyces* can enhance plant growth while suppressing disease using different mechanisms. *Streptomyces* sp. promotes plant growth by the production of plant growth hormones and siderophores, nitrogen fixation, and mineral solubilization, especially phosphates [16]. *Streptomyces,* with both antagonistic activity against pathogens and growth promoting ability, are attractive for development as a biocontrol agent to replace chemical fertilizers and pesticides in agriculture [21,27,28]. 

Biocontrol bacteria inhibit plant pathogens via several mechanisms, such as the production of antimicrobial compounds (antibiosis), iron sequestration (siderophores), production of extracellular enzymes that interfere with cell wall synthesis (chitinases, cellulase, proteases) and induction of plant-resistance mechanisms [12,16,23]. Rhizosphere *Streptomyces* in this study showed antagonistic activity against the rice blast fungus, *Pyricularia* sp., which correlated with their ability to produce siderophores, hydrolytic enzymes and antibiotics (Table 2). All actinomycete isolates were able to grow on CAS agar in the present study, which indicated their ability to produce siderophores. Siderophore-producing bacteria suppress some soil-borne fungal pathogens through iron competition [1,27]. All tested actinomycetes were also found to produce HCN. The production of HCN in excess may play a critical role in the control of fungal disease [29]. Microbial production of HCN has been suggested as an important antifungal activity to control root pathogens. Cyanide acts as a general metabolic inhibitor to avoid predation or competition [28]. The rice seedlings are not harmfully affected by inoculation with HCN-producing strains and the selected *Streptomyces* can act as a biological control agent. It is clear from the present investigation that rhizosphere *Streptomyces* sp. are able to produce antifungal substances as all isolates showed antagonistic activity against *Pyricularia* sp., though the active compound has yet to be purified and characterized (Table 2). Different levels of disease suppression in rice seedlings might be due to the different colonization pattern and secretion of secondary metabolites and/or cell-wall-degrading enzymes by the antagonistic bacteria. In the present study, all selected *Streptomyces* strains were found to colonize well in the rhizosphere of rice (Table 4). *Streptomyces* strain PC 12 was found to persist better than the other strains, and this might be the reason for the better performance in controlling blast disease. 

The cultural and morphological characteristics assigned the selected strain under the genus *Streptomyces*. Chemotaxonomic characteristics indicated that they belonged to genus *Streptomyces,* as the cell wall contained *LL*-diaminopimelic acid (cell wall type-I). BLAST analysis indicated that strain PC12 was closely related to *Streptomyces palmae* CMU-AB204^T^ with 98.82% similarity. It is recommended that 98.65% 16S rRNA gene sequence similarity can be used to differentiate between closely related species [30]. Therefore, in this study, the potent *Streptomyces* strain PC 12 was identified by 16S rRNA gene sequence analysis as *Streptomyces palmae* PC 12. However, from the position of strain PC12 in the phylogenetic tree, it formed a separate branch to its closest neighbor, *S. palmae* CMU-AB204^T^ (Figure 5). It is likely that strain PC12 may represent a novel *Streptomyces* species. However, a detailed polyphasic taxonomic characterization including whole genome sequence is required to confirm its status, which is clearly not the objective of the current study.

The results from this in vitro and greenhouse study suggest that the actinomycete isolates have potential to be used as biocontrol agents for the inhibition of rice blast disease by *Pyricularia* spp. 

## 4. Materials and Methods 

### 4.1. Isolation of Rhizospheric Actinomycetes

The isolation of actinomycetes was performed by conventional serial dilution spread-plate technique. The suspension from an appropriate dilution was inoculated on Actinomycetes Isolation Agar (AIA, Difco) and incubated at 30 °C for 7 days. The AIA medium was supplemented with 40 μg/mL of cyclohexamide to inhibit fungal growth and 10 μg/ml of nalidixic acid to inhibit other bacterial growth without affecting the actinomycetes [31]. Pure cultures were obtained by re-streaking on AIA medium and were stored as spore suspension in 20% glycerol for long-term preservation at -80 °C.

### 4.2. Isolation of Rice Blast Disease Fungi

Five infected rice plants (RD 6) were collected from the rice field in Maerim district, Chiang Mai province, Thailand (18° 56′ 17.2104” N and 98° 53′ 1.7520” E). Leaves with disease symptoms were cleaned under running tap water and cut into 1 × 1 cm segments. Surface sterilization was done by washing with 70% ethanol for 5 min followed by five rinses in sterile distilled water. Leaves were placed on potato dextrose agar (PDA) and final rinsing water was spread onto PDA medium to check the effectiveness of surface sterilization. The absence of microbial growth on the PDA medium confirmed that the surface sterilization procedure was effective in removing the surface bacteria [32]. Inoculated plates were incubated at 30 °C for 5 days. The fungal colony growing out from plant tissues was transferred onto fresh PDA medium. Pure cultures were observed under light microscope and were examined macro- and microscopically to identify the fungus as *Pyricularia* spp [25,26]. 

### 4.3. Pathogenicity Assay

Pyricularia sp. isolate was incubated on PDA medium and incubated at 28 °C for 5 days. The 14 day old plantlets at two-leaf growth stage were inoculated with 5 mm fungal plugs of Pyricularia sp. culture. The plugs were dropped on four leaves and wrapped with cotton moistened with distilled water. Humidity and moisture were maintained through the use of aluminum foil. The disease symptoms appeared 72 hours post-inoculation (hpi) and leaves were harvested [33].

### 4.4. In vitro Antifungal Assay

All the isolated actinomycetes were tested in an antagonistic assay conducted using the dual-culture technique. A 5 mm agar plug of *Pyricularia* sp. was placed on a side of the plate and another 5 mm agar plug with an actinomycete was placed on the opposite side [34]. Plates were incubated at 28 °C for 5 days and the antagonistic activity was scored according to the scale developed by Alfredo and Aleli (2011) [35]. The diameter of the mycelium growing out from the plug was measured and reported as the percentage inhibition of radial growth (PIRG) using the following formula [36]

PIRG = R 1-R 2/R 1 × 100

where R 1= Radial growth of *Pyricularia* sp. (mm) in the control plates

R 2 = Radial growth of *P*. *oryzae* interacting with antagonistic bacteria (mm)

Three biological replicates were performed and an average was taken.

### 4.5. Actinomycetes Identification

#### 4.5.1. Cultural and Morphological Characterizations

Morphological characteristics of the selected strain, including spore size and surface ornamentation, were assessed by compound microscope and scanning electron microscope (SEM) of 10 day old cultures grown on ISP-2 agar. The color of aerial spore mass, substrate mycelium and diffusible pigments of the selected strain were recorded after incubation at 30℃ for 10 days on ISP (International *Streptomyces* Project) medium (Difco) [37]. All characteristics were compared with Nonomura’s key [24]. 

#### 4.5.2. Cell Wall Composition Analysis

In order to determine the genus of antagonistic actinomycetes, the 2,6- diaminopimelic acid, one of the cell wall components of actinomycetes mycelia was analyzed using the method of Hasegawa et al. (1983) [38]. The selected strain was cultured in ISP-2 medium (4.0 g yeast extract, 10.0 g malt extract, 4.0 g dextrose, and 1 L sterile distilled water, pH 7.3) at 150 rpm, 28℃ for 14 days. After cultivation, the culture broth was centrifuged to collect cells. 6N HCl was used to hydrolyze the cells by heating at 70ºC for 18 h in a water bath. The hydrolysate was filtered through Whatman No.1 filter paper and evaporated to dryness in order to remove the HCl residue. Dried hydrolysate was dissolved in 1 mL of distilled water and applied onto TLC plate (15 x 20 cm, Merck Co., USA). A total of 20 ul of 0.01 M *DL*-DAP (Sigma Chemical Co., USA) containing both *meso*- and *LL*-DAP isomers and amino acids (alanine, glycine and glutamate) was also loaded on the TLC plates as a standard.

#### 4.5.3. Molecular Characterization of Potential Antagonistic Isolate 

The sequence of 16S rDNA of potential strain was determined after genomic DNA extraction and PCR amplification using eubacterial 16S rRNA gene-specific primers 27F (5’-AGT TTG ATC CTG GCT CAG GAC GAA CG-3’) and 1525R (5’-AGC CGG TCC CCC TGC AAG-3’) [21]. PCR amplification was carried out in 20 µL reaction mixture and amplification cycles, as described previously [27]. Amplified DNA was purified using a polymerase chain reaction (PCR) purification kit (Promega, Madison, USA). The sequencing was performed by commercial service of Pacific Science Co., Ltd., Thailand. A comparison of the obtained sequence with related *Streptomyces* strains in EzBiocloud database was performed using the Basic Local Alignment Search Tool (BLAST). The obtained 16S rRNA gene sequence was aligned with nucleotide sequences of related *Streptomyces* species, and the phylogenic tree was constructed by the neighbor-joining method using a MEGA X software package [39]. The resultant tree topology was evaluated by bootstrap analysis of the neighbor-joining data, based on 1000 resampled datasets [40]. The evolutionary distances were computed using the Maximum Composite Likelihood method [41] and are in the units of the number of base substitutions per site. This analysis involved 13 nucleotide sequences. Codon positions included were 1st+2nd+3rd+Noncoding. All ambiguous positions were removed for each sequence pair (pairwise deletion option). There were a total of 1339 positions in the final dataset.

#### 4.5.4. Scanning Electron Microscopy

Spore surface ornamentation was observed by scanning electron microscopy (SEM). Mycelia were taken after 10 days culture and washed in 0.1 M sodium cacodylate buffer (pH 7.4). They were fixed in 2.5% gluraraldehyde in 0.1 M sodium cacodylate buffer for 4 h at 4 °C, followed by post-fixation with 1% osmium tetraoxide in 0.1 M sodium cacodylate buffer (pH 7.4) and dried in a critical point dryer (EMITECH model K850, Hitachi). The specimens were mounted onto aluminium holders, sputter-coated with 10 nm Au and observed by SEM (Hitachi model S3400 at 15-30 kv, 2-5.00 μM).

### 4.6. Screening of Plant Growth Promoting Activities 

#### 4.6.1. Siderophore Production

Siderophore production was assayed on Chrome Azurol S blue agar (CAS) [42] and overnight bacteria culture were spotted on CAS plates and incubated at 30 °C for 5 days in the dark. The cultures showing a yellow to orange colored zone around colonies indicated siderophore production.

#### 4.6.2. HCN Production

Actinomyctes were streaked into ISP-2 agar supplemented with glycine. The plates were inverted and a piece of filter paper impregnated with 0.5% picric acid and 2% sodium carbonate was placed on the lid. After incubation for a week at 30 °C, the color change of the filter paper from yellow to orange was an indicator for HCN production [28].

#### 4.6.3. Hydrolytic Enzyme Production

Cellulase Production

Tested strains were grown on CMC agar for 5 days, flooded with 1% Congo red solution and washed with distilled water. The clear zone around the colony was observed and measured [28].

Chitinases Assay

Screening for chitinase production was done by agar plate assay on colloidal chitin medium containing 1.5% colloidal chitin, yeast extract 0.5 g, (NH_4_)_2_SO_4_ 1 g, MgSO_4_ 6H_2_O 0.3 g, KH_2_PO_4_ 1.36 g, agar 15 g and distilled water 1000 mL. The plates were incubated for 5 days at 30 °C and a clear zone around the colony indicated chitinase activity [28].

Proteases Assay

Protease activity was screened on skim milk agar containing (per liter): 5 g pancreatic digest of casein, 2.5 g yeast extract, 1 g glucose, 7% skim milk solution and 15 g of agar (pH 7.0). *Streptomyces* plug (6 mm) were placed on the medium and incubated at 30 °C for 5 days. Proteolytic activity was identified by the clear zone around the cell [28].

### 4.7. Greenhouse Experiments

The effect of the antagonistic Streptomyces sp. on controlling rice blast in vivo was assayed in greenhouse conditions. Rice (Oryza sativa L.) cultivar RD6 was used as a host plant as it is particularly susceptible to rice blast disease. The spore suspension of antagonistic actinobacterium (10^6^ CFU/mL) and the conidial suspension of Pyricularia sp. (10^5^ spores/mL) were prepared the same as for the in vitro assay. 

In greenhouse conditions, surface-sterilized rice seeds (RD 6) were sown in pots containing autoclaved soil (soil: sand; 3:1). The spore suspension of the antagonistic actinobacterium containing 0.2% (v/v) Tween 20 was sprayed on 10 day old rice leaves using a spray bottle (50 mL spore suspension per 100 seedlings). The rice leaves were sprayed with water containing 0.2% (v/v) Tween 20 as a control. The conidial suspension of Pyricularia sp. was sprayed 7 days later in the same way as described for the antagonistic actinobacterium. High humidity was maintained by the constant spray of moisture and the plants were kept under polythene shading. The experiment was carried out in pot culture with eleven treatments in four replications, following a completely randomized design (CRD). The treatments were prepared as shown below:

T 1 = Healthy control (no fungus, no actinomycetes);
T 2 = Healthy rice plant inoculate with *Streptomyces* strain W 1;
T 3 = Rice plant inoculate with *Streptomyces* strain W 1+ *Pyricularia* sp.;
T 4 = Healthy rice plant inoculate with *Streptomyces* strain D 4.3;
T 5 = Rice plant inoculate with *Streptomyces* strain D 4.3 + *Pyricularia* sp.;
T 6 = Healthy rice plant inoculate with *Streptomyces* strain PC 12;
T 7 = Rice plant inoculate with *Streptomyces* strain PC 12 + *Pyricularia* sp.;
T 8 = Healthy rice plant inoculate with *Streptomyces* strain D 4.1;
T 9 = Rice plant inoculate with *Streptomyces* strain D 4.1+ *Pyricularia* sp.;
T 10 = Disease control (*Pyricularia* sp. alone);
T 11 = Rice plant inoculate with Fungicide + *Pyricularia* sp.

The disease severity was evaluated 50 days after inoculating with *Pyricularia* sp. according to the standard evaluation system for rice [43]. The disease level was scored on a scale from 0 to 9 as follows:

0. no lesions observed;
1. small brown specks of pin-point size without sporulating centre;
2. larger brown specks but less than 1 mm in diameter;
3. small roundish to slightly elongated, necrotic grey spots 1– 2 mm;
4. typical susceptible blast lesions (spindle-shaped) 3 mm or longer, infecting less than 4.0% of the leaf area;
5. typical blast lesions infecting 4.1–10.0% of the leaf area;
6. typical blast lesions infecting 10.1–25.0% of the leaf area;
7. typical blast lesions infecting 25.1–50.0% of the leaf area;
8. typical blast lesions infecting 50.1–75.0% of the leaf area;
9. typical blast lesions infecting more than 75.1% of the leaf area.

The disease severity was computed using the following equation

$$Disease\;severity\;(\%)=\frac{Sum\;of\;disease\;grade\;\times \;No.\;of\;i\text{​}nfected\;tiller}{No.\;of\;tiller\;\times \;m\text{​}a\text{​}xi\text{​}mun\;disease\;grade\;\times \;No.\;of\;tiller}$$

The experiment was repeated twice with three replicates per treatment and 100 plants per replicate.

### 4.8. Effect of Streptomyces Application on Plant Growth Promotion under Greenhouse Conditions

#### 4.8.1. Measurement of Shoot and Root Weight and Tiller Number

The plants were uprooted carefully from the pot 60 days after transplantation. Tiller numbers were obtained. The root region was cut, separated from the plants and washed thoroughly to remove adhered soil particles. The fresh shoot and fresh root were dried by hot air oven at 50℃ for 48 h. The dry shoot weight and dry root weight of the plants of each treatment were measured in grams. 

#### 4.8.2. Rhizospheric Colonization by *Streptomyces*

Root apex samples from rice plants inoculated with each tested actinomycetes strains were taken 40 days after inoculation to check for rhizospheric colonization of bacteria. Root extracts and serial dilutions of 10^-3^-10^-5^ were made for the selection of bacteria. For both types of samples, 0.1 ml of each dilution was placed on ISP-2 medium and incubated at 30ºC for 72 h or until colony development was observed.

### 4.9. Statistical Analysis

The data were subjected to analysis of variance using SPSS 14.0 software. Mean values among treatments were compared by the least-significant difference (LSD) test at 5% level (*P* ≤ 0.05) of significance and presented as the mean values ± standard deviation (SD).

## 5. Conclusions

It is evident that selected rhizosphere actinomycetes successfully suppressed or reduced disease symptoms under both in vitro and in vivo greenhouse conditions. *S. palmae* PC 12 significantly reduced the disease severity by 56%. This strain also significantly increased plant growth attributes in infected plants. The results obtained in this study show the potential of *Streptomyces palmae* PC 12 as a biocontrol agent against rice blast fungus, *Pyricularia* sp., as well as in promoting the growth of rice variety RD6. The fungal inhibitory effect could be due to the production of cell-wall-degrading enzymes, such as chitinase, and HCN production. However, additional field trials are required to confirm the feasibility of *S. palmae* PC12 as an inexpensive, safe, and sustainable biopesticide for blast disease management.

## Figures and Tables

**Table 1:** Morphological characterization of one-hundred and twelve *Streptomyces* isolated from the rhizospheric soil of rice plant.

No.	Color of Aerial Mycelium	Soluble Pigment	Number of Isolates	Percentage of all Isolates (%)
1	Gray	Brown	41	36.6
2	Brown	Brown	23	20.5
3	White-Gray	Brown	14	12.5
4	White	Brown	12	10.7
5	Yellow	-	10	8.9
6	Green	-	3	2.7
7	Orange	Orange	3	2.7
8	Red	Brown	3	2.7
9	Pink	-	2	1.8
10	Black	-	1	0.9
	**Total**		**112**	**100**

**Table 2:** Plant growth promoting and antifungal activities of rhizosphere *Streptomyces* spp.

Isolate.	Inhibition Zone (mm)					HCN Production
	Antagonistic to *Pyricularia* sp. (%)	Siderophore Production	Protease Production	Cellulase Production	Chitinase Production	
PC-1	39.5 ± 2.7 f	11.4 ± 0.9 e	51.3 ± 1.9 b	-	-	+
PC-5	41.3 ± 1.8 e	8.9 ± 0.7 f	50.6 ± 1.5 b	-	-	+
PC-7	29.7 ± 3.5 g	10.4 ± 0.5 e	40.7 ± 1.1 c	-	-	+
PC-9	22.6 ± 2.9 g	12.5 ± 0.9 e	43.2 ± 1.3 c	-	-	+
PC-12	87.3 ± 1.8 b	24.5 ± 0.9 d	25.7 ± 1.3 e	57.2 ± 3.1 c	40.3 ± 2.7 c	+
PC-17	45.6 ± 3.1 e	18.5 ± 1.1 e	28.6 ± 1.5 e	43.1 ± 2.7 d	35.7 ± 2.1 d	+
PC-23	31.2 ± 4.9 f	17.3 ± 1.3 e	33.5 ± 1.3 d	27.9 ± 1.7 e	-	+
PC-24	71.6 ± 1.4 bc	32.4 ± 1.3 c	73.7 ± 1.5 a	-	-	+
PC-37	38.4 ± 7.8 f	28.9 ± 1.5 d	38.4 ± 1.7 d	-	-	+
PC-39	51.7 ± 1.9 d	17.5 ± 1.1 e	34.7 ± 1.5 d	-	-	+
PC-42	66.0 ± 3.5 c	32.8 ± 1.7 c	46.2 ± 1.1 c	-	-	+
PC-47	33.5 ± 3.1 f	36.7 ± 1.9 c	51.5 ± 1.9 b	40.5 ± 3.7 d	21.4 ± 1.3 e	+
PC-55	27.7 ± 7.5 g	41.0 ± 1.7 b	44.3 ± 1.7 c	64.2 ± 2.7 b	27.9 ± 2.7 e	+
PC-63	69.3 ± 3.7 c	40.5 ± 1.3 b	52.4 ± 1.5 b	-	-	+
PC-71	21.0 ± 1.7 h	34.6 ± 1.1 c	47.2 ± 1.9 c	-	-	+
PC-73	38.6 ± 2.1 f	43.4 ± 1.3 b	24.2 ± 1.3 e	-	-	+
PC-88	26.1 ± 0.8 g	42.8 ± 1.5 b	35.7 ± 1.7 d	-	-	+
PC-94	71.3 ± 1.4 bc	50.2 ± 1.3 a	28.9 ± 1.5 e	-	-	+
PC-102	38.3 ± 4.0 f	34.5 ± 1.5 c	47.6 ± 1.3 c	57.6 ± 4.1 c	-	+
PC-110	49.5 ± 2.3 e	37.4 ± 1.3 c	28.7 ± 1.7 e	-	-	+
PC-117	67.2 ± 4.2 c	50.7 ± 1.1 a	37.8 ± 1.9 d	-	-	+
PC-119	75.8 ± 3.7 b	40.8 ± 1.5 b	45.4 ± 1.5 c	-	-	+
PC-120	74.3 ± 4.2 b	31.7 ± 1.3 c	50.2 ± 1.9 b	-	-	+
D 4.1	82.2 ± 2.5 a	52.3 ± 1.9 a	53.5 ± 1.9 b	70.8 ± 4.7 a	57.4 ± 3.1 a	+
D 4.3	80.0 ± 2.7 a	51.4 ± 1.1a	52.2 ± 1.9 b	69.5 ± 3.7 b	48.5 ± 2.7 b	+
W 1	80.5 ± 1.7 a	47.8 ± 1.5 c	50.7 ± 1.5 b	57.8 ± 1.9 c	34.8 ± 2.1 d	+
W 9	76.4 ± 3.2 b	41.5 ± 1.1 c	34.7 ± 1.3 d	-	-	+

“Positive” (+): Having trait; “Negative” (-): Not Having trait. Data are means ± standard error over four replicates. Numbers in column followed by the same letter are not significantly different according to Duncan’s multiple range test (DMRT) at *P* ≤ 0.05.

**Table 3:** Effect of selected *Streptomyces* application on blast disease and plant growth promotion under greenhouse conditions.

Treatment	Blast disease Parameters 60 Days			Plant growth Parameters60 Days			
	Lesion Length (cm)	No. of Dried Leaves per Plant	Disease Severity (%)	Plant Height (cm)	Root Length (cm)	Root Dry Weight (g)	No of Tiller
*Streptomyces* W1	1.19 ± 0.21 b	3.67 ± 0.27 c	51.9 ± 0.31 c	45.08 ± 0.57 c	29.43 ± 0.17 c	2.13 ± 0.07 ab	12.8 ± 0.31 bc
*Streptomyces* D 4.3	0.98 ± 0.19 b	3.37 ± 0.21 c	44.5 ± 0.37 b	52.69 ± 0.47 b	37.00 ± 0.19 b	2.53 ± 0.11 a	15.2 ± 0.27 b
*Streptomyces* PC 12	0.57 ± 0.19 c	3.10 ± 0.19 c	31.4 ± 0.37 a	76.93 ± 0.59 a	41.60 ± 0.27 a	2.68 ± 0.17 a	19.4 ± 0.45 a
*Streptomyces* D 4.1	1.07 ± 0.15 b	4.50 ± 0.29 b	42.8 ± 0.31 b	43.57 ± 0.37 c	31.89 ± 0.21 b	2.05 ± 0.19 ab	14.7 ± 0.21 b
Pathogen (treated control)	6.78 ± 1.17 a	8.38 ± 0.35 a	87.5 ± 0.47 d	36.59 ± 0.41 d	26.17 ± 0.15 c	1.88 ± 0.13 b	ND
Healthy (Untreated control)	ND	ND	ND	40.70 ± 0.35 cd	29.43 ± 0.29 c	1.96 ± 0.17 b	9.20 ± 0.31 d

ND = Not detectable; data are means ± standard error over four replicates. Numbers in column followed by the same letter are not significantly different according to Duncan’s multiple range test (DMRT) at *P* ≤ 0.05.

**Table 4:** Cell viability of selected *Streptomyces* sp. at 60 dpi.

Streptomyces Strains	Cell Number (CFU/mL)
Streptomyces W1	3.2 × 10^3^
Streptomyces D 4.3	3.4 × 10^4^
Streptomyces PC 12	4.5 × 10^4^
Streptomyces D 4.1	2.7 × 10^3^
